# Elephant (*Elephas maximus*) Health and Management in Asia: Variations in Veterinary Perspectives

**DOI:** 10.1155/2015/614690

**Published:** 2015-01-19

**Authors:** David Miller, Bradford Jackson, Heidi S. Riddle, Christopher Stremme, Dennis Schmitt, Thaddeus Miller

**Affiliations:** ^1^P.O. Box 2786, Loveland, CO 80539-2786, USA; ^2^University of Alabama at Birmingham, Birmingham, AL, USA; ^3^Riddle's Elephant and Wildlife Sanctuary, AR, USA; ^4^Elephant Health Care Program (EHCP) of the Veterinary Society for Sumatran Wildlife Conservation (Vesswic), Sumatra, Indonesia; ^5^William H. Darr School of Agriculture, Missouri State University, Springfield, MO, USA; ^6^Ringling Bros. Center for Elephant Conservation, Polk City, FL, USA; ^7^University of North Texas Health Science Center, Fort Worth, TX, USA

## Abstract

There is a need to identify strategic investments in Asian elephant (*Elephas maximus*) health that will yield maximal benefits for overall elephant health and conservation. As an exploratory first step, a survey was administered to veterinarians from Asian elephant range countries at a workshop and via email to help prioritize health-related concerns that will mostly benefit elephants. Responses were received from 45 veterinarians from eight countries that had a range of experience with captive and wild elephants. The occurrence of medical conditions and responses to treatment varied among responses. However, injuries, parasitism, and gastrointestinal disease were reported as the most common syndromes responsible for elephant morbidity, whereas injury and infectious disease not due to parasitism were the most commonly reported sources of elephant mortality. Substandard nutrition, water quality and quantity deficiencies, and inadequate or absent shelter were among the factors listed as barriers to optimal elephant health. While this survey's results do not support definitive conclusions, they can be used to identify where and how subsequent investigations should be directed. Rigorous assessment of the relative costs and benefits of available options is required to ensure that investments in individual and population health yield the maximal benefits for elephants.

## 1. Introduction

Wildlife conservation, veterinary and public health, and other organizations allocate human, physical, and economic resources according to perceived needs and available resources [1–3]. The allocation decision process can be complicated, such as where investment in animal health does not complement conservation objectives and vice versa. Baseline information on where and how to direct and integrate efforts for animal health and conservation is generally incomplete and sometimes absent when organizations make resource allocation decisions. Consequently, exploratory work needs to be conducted to clarify the primary issues of concern. This approach is not unlike traditional public health and veterinary population health programs where problems are identified, and the costs and benefits of interventional strategies are established, as a part of overall program development and prioritization of resource allocations [3, 4].

Asian elephants (*Elephas maximus*) are an endangered species that illustrate the challenges of optimizing and integrating resource allocations for animal health and conservation [5]. Across their range, Asian elephants face conflict with humans, habitat destruction and fragmentation, diseases, and other challenges [6]. Wild elephant populations may be adversely or positively affected by captive populations, which have limited fecundity and undocumented levels of compromised health. Addressing these and other challenges with a unified and appropriate policy or practice is complicated because there are many differences in the culture, attitudes, and infrastructure experienced by elephant personnel located in different Asian elephant range countries. Consequently, what should take priority in efforts to sustain wild and captive elephant populations is unclear. To catalog the collective experience and skills among elephant veterinarians in Asia is a necessary early step to more clearly understand existing challenges and more effectively design strategies that will reverse or slow trends that threaten elephant health and population viability.

We report findings from a review of the published literature to document the types of studies and topics relevant to elephant health and conservation in Asian elephant range countries. This is followed by the results of an initial survey of veterinarians across Asian elephant range countries. Our survey was by necessity conducted in a nonrandom, semiformal fashion and cannot be interpreted as fully representative. It can, however, begin to lay the foundations for more comprehensive work by both using a new approach and identifying knowledge gaps critical to the appropriate design and successful conduct of subsequent work. Because management practices and their effects may ripple between wild and captive elephant populations, we considered the overall conservation of Asian elephants by systematically collecting data related to common elephant health concerns from veterinarians with elephant expertise working in Asia. We used a survey instrument that included questions concerned with background information, basic veterinary concerns, laboratory facilities, elephant handler (mahout) health, perceived needs for improved elephant health, and common diseases or conditions. This provides preliminary information that supports methods development and hypothesis generation for subsequent work to improve elephant health and conservation programs in Asia.

## 2. Methods

### 2.1. Literature Review

We conducted a search of the PubMed database (http://www.ncbi.nlm.nih.gov/pubmed) using the terms “elephant” and “Asia” (26 August, 2014). Titles, author address, and abstracts (where available) were reviewed to establish whether the research was conducted in Asian elephant range countries; references that clearly were not from these countries were excluded. Studies were characterized, where possible, by study design (laboratory, controlled trials, hybrid, case report or series, cross-sectional surveys, case-control, cohort study, policy, review, hybrid, and unable to determine) and topic categories (basic veterinary concerns, laboratory capacity, needs assessments, common disease conditions, and treatment).

### 2.2. Survey Development

We developed and administered a 13-page, 21-field survey instrument to explore the veterinary knowledge and practices of a convenience sample of veterinarians from Asian elephant range countries (see the Appendix). The English language instrument included closed- and open-ended questions to collect data in 6 areas: (1) basic background information (country, employment, elephant caseload, and the elephant population's characteristics); (2) basic veterinary concerns (prevalence of various clinical conditions); (3) laboratory facilities (availability of various laboratory services); (4) informal impressions of mahout health (clinical conditions observed and knowledge of routine screening); (5) needs assessment (clinician's needs for medicine, treatment, diagnostics, basic husbandry, and mahout training that could improve elephant health); and (6) common disease conditions (ranked medical conditions, treatment, and outcomes). Questions were developed to gain insights into broad, coarse-scale trends in elephant health and serve as a starting point for where to direct subsequent, more focused investigations. The survey was drafted in consultation with veterinarians with expertise in elephant health and others, including a biostatistician and a biomedical systems researcher, in order to provide the most comprehensive, relevant, and valid information available given the constraints of circumstance, language, and practice. The survey was pilot tested for ease of readability and completion.

### 2.3. Study Population

Data was collected from two populations: a convenience sample of veterinarians participating in an elephant health workshop (Regional Asian Elephant Veterinary Workshop held in Banda Aceh, Sumatra, Indonesia from March 27–30, 2012) and a convenience sample of veterinarians that were subsequently contacted via networking and responded via email. Potential participants were excluded if they had insufficient written English language proficiency to consent to participate in or complete the survey. All respondents were offered the opportunity to respond anonymously. The study population represented a broad spectrum of involvement in elephant health and varying levels of experience.

All veterinarians attending the workshop who consented to participate were administered the survey during the first morning's session, prior to any presentations that could influence responses. Surveys were collected immediately after completion and subsequently scanned to create permanent electronic records. Emailed survey responses were solicited from veterinarians that were known but unable to attend the workshop.

Prudent and conservative research practice requires that when planning studies and recruiting study participants, the potential harm to participants must be minimal and fully disclosed. In keeping with these standards, our methods and survey instruments were reviewed and approved as appropriate by the University of North Texas Health Science Center (Fort Worth, Texas, USA), Office for the Protection of Human Subjects, Institutional Review Board.

### 2.4. Data Analysis

Data from surveys was entered into electronic format by one investigator (B.J.) and verified by a second investigator (D.M.). In an attempt to compensate for uneven English language skills, we used a web-based program (Google Translate; https://translate.google.com/) or a native translator. Where this strategy allowed survey completion by workers with otherwise insufficient English language proficiency, those responses were included; where language barriers precluded interviewees from giving consent or substantially completing the survey in spite of translation attempts, the responses were not included.

Except where marked differences were noted, data from responses obtained from workshop and email responses and data from respondents working with captive and wild elephants were combined for analysis. This was done for the purpose of obtaining sufficiently large sample sizes to gain insights into the broad issues of interest to this study, rather than focus on precision or outliers. In addition, responses from those that worked with both captive and wild populations often did not distinguish between the two populations. The appropriateness of this approach was qualitatively confirmed by examining the data for substantial differences between the sample populations. Descriptive analyses were conducted; categorical responses are presented as absolute numbers and percentages and continuous responses are presented as means ± standard deviations (SD). As a means of accounting for the diversity of responses and limited diagnostic capacity available to clinicians, observed medical conditions were qualitatively grouped into syndromes and reported by rank; syndrome scores were not reported to avoid inferences that were not appropriate based on the convenience sampling scheme.

## 3. Results

### 3.1. Literature Review

The literature search identified 202 references from 1963 to present. Many references had limited information available, but 70 were identified as relevant. The most common study designs were ecological/natural history, case reports or series, and laboratory-based investigations that documented physiological or biomedical reference ranges and measurements (Table 1). The most common study topics were descriptions of common disease conditions. Of 44 reports that could be identified as being conducted in a single country, over half were conducted in India (*n* = 15) and Sri Lanka (*n* = 10).

### 3.2. Survey Overview

Responses were obtained from 45 individuals from a total of 8 Asian elephant range countries for a total of 8595 data points; 15 of these data points were not legible or could not be translated and were excluded (Table 2). Most (*n* = 27, 60%) respondents were government employees. Over half of the respondents worked with elephants full-time (*n* = 24, 53%), with the remainder having part-time (*n* = 11, 24%) or exclusively academic responsibilities. The duration of relevant experience among respondents ranged from 0.5–30 years (mean 10.7, SD ± 6.8 years).

The mean number of elephants checked per month by government (23.1 SD ± 27.4) and nongovernment (26.6 SD ± 30.5) veterinarians was similar but varied widely by individual respondent (occasional to 100 elephants examined per month) as did the reported number of elephants seen at least twice per year (occasional to 3000 elephants). Fewer (11% versus 42%) respondents reported their primary work (80–100% of contacts) to be with wild rather than captive elephants.

None of the respondents indicated that all of the elephants in their area were reproducing, but 71% indicated that at least some elephants had successfully reproduced. Seven respondents did not provide responses regarding elephant reproduction. A variety of primary uses for elephants were indicated by respondents; responses of “all” or “most” indicated their use in: zoos (*n* = 7), tourism (*n* = 13), logging (*n* = 6), forest patrol (*n* = 7), conflict management (*n* = 2), or education (*n* = 3).

### 3.3. Basic Veterinary Concerns

Most elephants that were regularly examined had some medical abnormalities; few respondents (14%) indicated that 80–100% of elephants that were regularly checked had no visible signs of disease. Reported conditions that together comprised a small percentage of the total “abnormal” report included evidence of systemic illness, upper or lower respiratory disease, weight loss, diarrhea or other gastrointestinal problems; ectoparasites; nonectoparasitic skin disease; endoparasites observed in feces; eye disease; oral disease, tusk or molar disease; trunk disease; foot disease or other sources of lameness; wounds other than skin lesions; infant mortality; reproductive problems; reduced work endurance; and anorexia. Responses were not uniform within or between countries. Gastrointestinal disease was not confined to captive animals, as two respondents with a focus on wild populations indicated that 40–59% of their elephants had colic and/or diarrhea. Regional differences were reported for nonparasitic skin diseases; half of Indian respondents indicated that 40–59% of elephants had nonparasitic skin diseases, whereas most (86%) respondents from Sri Lanka reported that nonparasitic skin diseases were uncommon (0–19% of regularly inspected elephants). Respondents from five countries reported that <40% of their elephants were shedding endoparasite ova or larvae (the number of responses from each country varied from 1 to 15). The highest prevalence (60–79%) of eye disease was reported for Indian elephants (38% of respondents). Oral, tusk, and molar disease was reported sporadically, with two respondents indicating that these conditions were observed in 40–59% of elephants, and one respondent indicating a prevalence of 60–79%. These oral conditions were not coreported with responses from three countries where anorexia was reported for 60–79% of elephants; most responses indicated that 0–19% of elephants had anorexia. Stereotypic behavior as common as 60–100% in some populations was reported, and stereotypies were not restricted to captivity, as three respondents reported 20–80% of free-ranging elephants with stereotypic behavior. Two workshop participants from Indonesia reported a prevalence of 20–39% for trunk disease, whereas the remaining responses indicated a prevalence of < 20%. The prevalence of simple foot disease was reported to be 20–60% by half of the respondents. Complicated foot disease was reported to be < 40% by 62% of the respondents, and three respondents indicated that > 20% of the free-ranging elephants had complicated foot disease. Only 13% of respondents reported that > 20% of elephants had infant mortality or reproductive problems.

Most (84%) of the respondents had conducted necropsies, at a mean of 5.37/year (SD ± 12.05) (range < 1 to 40/year). A variety of pathologic findings, including specific etiologies and specific organ lesions, were reported for necropsies (Table 3). However, no single condition exceeded 11% of the total. Trauma from all causes (human elephant conflict, intraspecific aggression, traumatic injuries, and lightening) comprised 18% of the reported findings, which exceeded the sum of infectious diseases (16%) due to parasitism, and infectious bacterial and viral diseases.

### 3.4. Laboratory Facilities Questionnaire

Hematology (56%), laboratory analyses for routine organ function (e.g., AST, alkaline phosphatase, BUN, creatinine, etc.) (51%), and light microscopy for fecal parasite testing (78%) were always or mostly available to over half of respondents (Table 4). Light microscopy for blood parasite testing was always or mostly available to 44% of respondents. Approximately one quarter of respondents had access to other diagnostic modalities, with 53–84% having no access to nutrition-related, virology, or imaging diagnostics. With the exception of blood mineral, blood nutrition, virology, and toxicology, respondents working primarily with captive animals had greater access to diagnostics.

### 3.5. Mahout Health Questionnaire

Only 29% of the respondents indicated that the health of mahouts was routinely screened, although another 20% were unsure of whether mahout health screens were conducted. Respondents mostly indicated that few (0–19%) mahouts had frequent sneezing or coughing, sudden weight loss, eye disease, abnormal joints or limbs, skin abnormalities, or lethargy. One respondent reported that 60–79% of mahouts had abnormal limbs, joints, or skin, and this individual also reported 40–59% had lethargy, eye disease, frequent coughing, and frequent sneezing.

### 3.6. Needs Assessment

Most respondents indicated that an important need is improvement in basic husbandry. More than 2/3rds of respondents indicated a need for improved supplements, and narrative responses indicated great interest (*n* = 32 responses, 71%) in improving the nutritional offerings provided to elephants. Most (64%) respondents indicated a need for improved elephant shelters, although some captive elephants resided in “deep forest” and were not considered in need of shelter. Many respondents listed a need for facilities for conducting treatments (56%) and managing adult bulls in musth (51%). Half (51%) of respondents had concerns for water quality and/or quantity. Narrative responses indicated concern for safe drinking and bathing water and a need for filtration and water quality testing. Most respondents indicated a need for improved mahout training in the areas of daily routine health (80%), elephant biology and behavior (64%), elephant restraint and handling tools (62%), and drug use and administration methods for elephants (60%).

Overall drug availability limitations (64%) were considered the greatest constraint for addressing elephant health needs (Table 5). The most common preventive medications administered included anthelmintics (*n* = 28), vaccination (*n* = 11), vitamins (*n* = 9), and minerals (*n* = 6). Antibiotics were also listed as preventive medications by a few respondents (*n* = 5). Respondents described multiple anthelmintics and regular rotations of drugs, and practical concerns such as short expiration times were reported. Routinely administered vaccinations included tetanus toxoid (*n* = 21), rabies (*n* = 14), hemorrhagic septicemia (*Pasteurella multocida*) (*n* = 6), foot-and-mouth disease (*n* = 6), anthrax (*n* = 4), blackleg/black quarter disease (*Clostridium chauvoei*) (*n* = 3), and tuberculosis (mycobacterial species and vaccine type not specified) (*n* = 1). Narrative responses indicated that vaccine availability and cost concerns limit the number of elephants that are vaccinated, and there was concern for the absence of elephant-specific vaccines. Narrative responses also elaborated on owner financial limitations and the inability of mahouts to continue drug and other treatments. Untrained or uncooperative elephants (36%) were commonly reported constraints for administration of medications and performing diagnostics. Additional recommendations for improved preventive health programs included a need to increase the number of knowledgeable veterinarians and mahouts.

The scarcity of diagnostics that could be the basis for optimizing treatments (67%) was listed as a common obstacle. Limited test availability (64%) and a shortage of trained personnel (51%) represented the greatest obstacles for performing diagnostic procedures. Narrative responses noted the absence of elephant-specific assays, limitations of existing technologies for such a large species, problems with storage of samples, levels of professionalism, and slow laboratory turn-around times.

### 3.7. Common Disease Conditions

The most common syndromes reported as causes of morbidity in range country Asian elephants were injuries, endo- and ectoparasitism, and gastrointestinal disease (Table 6). While many of the syndromes were reported by respondents that had either predominantly captive or wild responsibilities, injuries due to human-elephant conflict, as well as poisoning, were reported exclusively for wild populations, with one exception. Abscesses, cancer, nonparasitic infectious diseases (rabies, tetanus, anthrax, herpes, and tuberculosis), overwork, renal disease, reproductive problems, skin disease, stereotypical behavior, tusk pathology, and ventral edema were reported exclusively for captive elephants, although none of these were common. Lameness was also exclusively reported for captive elephants, although foot pathology was reported for both captive and wild populations. Infectious disease due to parasitic and nonparasitic causes had a cumulative percentage score (21%) that was less for injuries due to all causes (27%), although causes of gastrointestinal disease, ocular disease, skin disease, abscesses, foot pathology, and lameness could have had infectious disease or injury components that could not be established with this study. Of 14 respondents reporting bacterial or viral infections by name (elephant endotheliotropic herpes virus or EEHV, tuberculosis, anthrax, foot and mouth disease, tetanus, elephant pox,* Salmonella, Clostridium*, or rabies), only 3 mostly or always had access to either microbiology or virology laboratory facilities.

Injuries (35%) were ranked as the leading cause of mortality, followed by infectious disease not due to parasitism (14%). Gastrointestinal disease, poisoning, geriatric, nonspecific, and parasitic etiologies were ranked similarly as causes of mortality. Respondents from four different countries (India, Indonesia, Malaysia, and Sri Lanka) reported mortality due to poisoning, with similar numbers of reports from captive and wild populations. Organ-based disease (renal and cardiac) and malnutrition were similarly ranked less than 5% as a cause of mortality, followed by a number of miscellaneous causes. Gunshot, explosives, railway accidents, poaching, human-elephant conflict, and snares were reported primarily from Sri Lanka (71%) and wild populations. Poaching was also reported for respondents from India, Malaysia, and Indonesia. Traumatic causes of mortality reported for captive populations included intra-specific fighting, work accidents, lightning, and other injuries.

Nine syndromes (injuries, gastrointestinal disease, malnutrition, lameness, foot disease, infectious disease, parasitic disease, ocular disease, and miscellaneous) were subjectively identified among respondent's lists of standard treatments, success rates, and treatment needs, with recognition that these categories were not strictly distinct and did not represent formal diagnoses (Table 7). Subcategories for each syndrome were identified where treatment, and/or success rates, and treatment needs were distinct. A range of treatments, success rates with and without treatment, mortality, and clinician needs were reported, with variable numbers of reports for each category. Respondents also identified basic medical (e.g., suture), husbandry (e.g., elephant training for routine preventive care and improved housing), and diagnostic (e.g., clinical laboratory, diagnostic imaging) needs.

## 4. Discussion

This study represents the key first step of ensuring that the most important needs for elephant health and conservation in Asia receive the highest priorities [3]. Our literature review indicated that elephant health and conservation reports are predominantly descriptive with few efforts to address population-level concerns. Our interest in conducting the survey was in developing a new approach and identifying broad trends to serve as a starting point for identifying where more focused efforts are needed. By necessity, a convenience sampling scheme was used. Consequently, as with all observational studies, limited inferences are possible from this data. However, a broad overview of the data suggests that the health and conservation needs of elephants vary regionally. Even if this is not true, the differences among responses suggest regional variation in perspectives that must be considered when addressing elephant health and conservation needs. Differing perceptions of the questions and categories, the survey administration methods used, and other biases may limit confidence in the absolute numbers or relative ranks of syndromes and other observations. Thus, while this study could not resolve all questions, gaps in clarity and other sources of uncertainty can direct future efforts. This will increase the odds that strategic investments yield the maximal benefits for elephant conservation.

### 4.1. Literature Review

Our literature review indicated that published research on elephants in Asian range countries was primarily descriptive and was concentrated in a few countries. There have been efforts to survey elephant health in Asia [7]. However, there is a dearth of broad-scale studies that can guide researchers and funders by identifying prioritized needs that are most likely to have a beneficial impact on captive and free-ranging elephant health and conservation. While case reports and other descriptive studies can be useful for sharing valuable information, they reflect the interests and support for publication of the authors and are difficult to place in context per the impact of these conditions on elephant populations. Consequently, available literature is not sufficient for guiding strategic approaches for the use of limited resources to benefit elephant health and conservation in Asia.

### 4.2. Survey Overview

As with all questionnaires, recall and other biases, the format of closed questions, and other concerns are limitations [8]. As is common for conservation-related questionnaires, formal validation of the questionnaire, opportunistic sampling from a limited sampling frame, respondents' source of employment, and other concerns exist. Also, exclusion of non-English speaking veterinarians and those without access to the Web, as well as culturally-based misinterpretation of the questions, are among potential limitations of this study. In particular, biases are possible due to representation from only 8 of 13 Asian elephant range countries and overrepresentation of respondents from one country (Indonesia, where the workshop was held). In retrospect, inclusion of 0% and 100% categories may have eliminated some of the ambiguity associated with the ranges that the questionnaire provided. Efforts to improve the study's relevancy and scope included external review of the questionnaire's format by multiple professionals prior to distribution, increasing the sample size and the range of countries by distributing the questionnaire in person at a workshop and via electronic networking, offering the option of anonymous responses, and inclusion of respondents with primarily captive, primarily free-ranging, and mixed-setting elephant experience. Ultimately, while this study is the first effort to objectively investigate the health-related syndromes and needs of elephants in Asia, it serves as a coarse-scale starting point for optimizing resource allocation for this region's elephants.

### 4.3. Basic Survey Information

The small number of responses from some countries and for some categories, as well as incomplete responses that further limited the dataset, precluded formal statistical comparisons. The full sampling frame of English and non-English speaking elephant veterinarians in all 13 Asian elephant range countries could not be established. Qualitatively similar results for respondents that received the questionnaire via in-person and electronic networking resulted in joint summary of the results, except where noted. As many respondents did not strictly work with captive or free-ranging elephant populations, distinctions among captive and free-ranging elephants are limited. Although respondents could respond anonymously, 60% of respondents were government employees, the majority of elephants were owned by the government, and the degree to which government policy influenced responses is unclear.

### 4.4. Basic Veterinary Concerns

While most elephants were classified as having few visible signs of disease, substantial variation was observed among the responses and a number of different conditions were responsible for the total number of abnormalities reported. There is a need to establish whether differences in veterinary knowledge and/or training, elephants trained for examinations, support for diagnostic facilities, regional differences in disease, husbandry, individuals' perceptions, or other reasons can account for differences in reported conditions between and within countries. In particular, the cost effectiveness for improved husbandry and preventive care should be explored further. Each of the respondents (*n* = 9) indicating that >40% of regularly checked elephants had colic or constipation also indicated a need for improved food quality, access to medications, and the presence of endoparasites in >20% of elephants, and most also indicated a need for improved water quality. This data suggests that improved basic husbandry and routine prophylaxis may reduce the prevalence of these conditions and possibly others related to work endurance, fecundity, anorexia, and oral health. Alternatively, some interventions may be uncertain. For instance, only 54% of respondents reported routine use of anthelmintics in countries where fecal shedding of parasites was classified as uncommon, although limited diagnostic availability may be responsible for underdiagnosis of fecal shedding. Limited diagnostic availability also raises uncertainty as to whether agents, such as the parasite* Cobboldia elephantis* [9], are focally or widely distributed. Responses indicating foot disease and stereotypic behavior in free-ranging populations are of uncertain significance and may warrant further investigation for perspective on management of captive populations. Low fecundity reported in the Basic Information section of the questionnaire and few respondents reporting low infant mortality or reproductive problems warrant clarification of whether the reported infrequency of reproductive problems is real or the consequence of reproduction being a low management priority for these populations. An understanding of where clusters for some conditions truly exist, where diagnostic facilities or clinician training are limited, and how to facilitate increased training of mahouts and elephants for preventive medicine, diagnostics, and treatment is needed.

Most respondents conducted necropsies, although the need for improved equipment and the physical challenges were noted and might affect the accuracy of necropsy results. In addition, the likelihood that not all elephants are necropsied (for a variety of reasons) is a bias that limits population-level inferences. Trauma-related necropsy findings were marginally more common than the sum of mortalities due to all infectious causes. However, improved diagnostic capacity might result in different proportions of pathologic findings. The diversity of conditions noted by respondents indicates the need to identify the medical conditions that can be most cost-effectively managed to improve animal welfare and/or population stability or growth; focusing on one or a few diseases that are not strategically selected to ensure that resource investments have a substantive impact may not result in improved individual or population-level health and welfare.

### 4.5. Laboratory Facilities

Basic hematology and blood chemistries were always available to only 9% of the respondents. This is consistent with the absence of studies in our literature review that document the laboratory capacity for diagnosing elephant medical conditions in Asian elephant range countries. While light microscopy was available for fecal parasitology for most respondents, there is uncertainty why only 44% always or mostly also have access to light microscopy for identifying blood parasites. This could be because light microscope optics for evaluation of fecal ova are less (10–40X) than needed to evaluate the presence of blood parasites (100X oil immersion). Alternatively, this discrepancy may be linked to responses elsewhere in the questionnaire indicating a need for improved personnel training. Few respondents had access to histopathology all or most of the time. In light of this, the dearth of testing available for nutritional concerns, toxins, microbes, or diagnostic imaging is not surprising. These diagnostic limitations affect both antemortem and postmortem diagnoses. Consequently, many of the diagnoses listed in the questionnaire are likely based on clinical signs or gross pathology only. This limits inferences possible from this and other elephant health studies that are conducted in Asia, and therefore increases uncertainty about the prevalence and impact of various disease conditions on elephant health, welfare, and population viability. Better confidence in the diagnoses would require investment in improved laboratory facilities and trained personnel. This would likely improve antemortem diagnoses and clinical case management, as well as assessments of population health and disease.

### 4.6. Mahout Health Questionnaire

Mahout and elephant health may be closely linked by the mahouts' ability to care for their elephants, the elephants' ability to work, and other factors [10], and this potential link was not discussed in studies identified in our literature review. The informal observations of the veterinarians responding to this survey serve as a starting point for investigating this concept from the perspective of trained medical professionals.

Only six individuals from six countries reported that >19% of mahouts had any of the listed medical conditions. Possible reasons for these discrepancies include clusters of disease occurrence, a majority of respondents focusing their attention on elephants rather than mahouts, or other reasons. Addressing mahout health could result in improved elephant health by improving the mahouts' ability to provide care to their elephants or as part of a strategy to gain support for elephant-oriented health and welfare programs. Further investigations in this area are warranted to clarify this survey's findings.

### 4.7. Needs Assessment

Responses to both open and closed format questions indicated notable needs on several levels. Basic elephant husbandry needs reported by respondents included improved nutrition; elephant shelters and musth bull facilities; water quality and/or quantity; and mahout training. Respondents indicated that improved elephant care required better diagnostic capacity; facilities to perform treatments; trained personnel; trained and cooperative elephants; and improved drug and vaccine availability. The absence of reliable diagnostics limits the therapeutic value of many drugs due to uncertain diagnoses and also curbs confidence in the results of many studies of elephant disease.

Based on other species, the most cost-effective priorities for improving elephant health and welfare in Asia may be addressed via preventive medicine programs, basic husbandry, diagnostic capacity, prophylactic medications, and training of veterinarians and mahouts. As more than two thirds of all respondents indicated a need for improved nutrition, provision of an appropriate diet is a strategy that will theoretically support all other health objectives. Similarly, provisions of appropriate shelter and water are fundamental husbandry concepts that should be considered for programs intended to improve elephant health and welfare. Vaccines for rabies,* Clostridium chauvoei*, hemorrhagic septicemia (*Pasteurella multocida*), and tetanus could be cost-effective preventive measures, although efficacy and the relative cost-effectiveness of all vaccination options warrant further investigation. Also worthy of further investigation is the degree to which elephant health could be improved by increased use or appropriate rotation of anthelmintics and development of effective education programs for veterinarians and mahouts. Owner financial constraints or perceptions of the benefits of health measures may limit the potential to enact preventive and medical treatment programs. Conventional medical strategies are largely relevant to captive elephants. Broader scale studies that incorporate ecological and sociological factors are likely more relevant to improving the health of free-ranging elephants.

### 4.8. Common Disease Conditions

Injuries and parasitism were the most common medical conditions seen in captive and free-ranging elephants, with injuries due to human-elephant conflict restricted to free-ranging populations (Table 6). These medical conditions are candidates for preventive management in captive populations via administration of antiparasitic agents and husbandry modifications for minimizing intra-elephant conflict. Strategies for mitigating human-elephant conflict may reduce mortality in wild populations [11, 12]. Ocular disease due to eye worms and other causes was a comparatively common condition, although the response to treatment was often successful for captive elephants. While lameness was reported exclusively for captive elephants, recognition of foot pathology in free-ranging elephants warrants further investigation, as it has relevance to concerns for captive elephant management and welfare [13–15]. Gastrointestinal disease, including colic, bloat, and constipation, was common in captive animals and was also reported for wild populations. Although gastrointestinal disease represents a broad range of etiologies, the degree to which improved nutrition and access to safe water can reduce the prevalence of gastrointestinal disease and malnutrition for elephants is worth exploring further, especially in calves. Infectious disease was uncommonly reported, although the degree to which this represents deficiencies in access to diagnostic laboratories versus a legitimately low concern is uncertain. In particular, diseases such as EEHV and TB that garner much attention in nonrange, for example, North America and Europe, captive elephant populations are difficult to consider when many Asian countries do not appear to have mycobacterial and virologic veterinary testing capabilities sufficient for providing accurate diagnoses. Similarly, assessing many of the less common diseases is difficult in the absence of reliable diagnostic support. Nevertheless, questionnaire responses suggest that injuries may cause over one-third of mortalities, and this estimate may be low if abscesses or other conditions are secondary to injuries.

A range of responses to treatment was evident (Table 7). This could be because combining narrative reports into consolidated categories such as injuries, gastrointestinal disease, lameness, and foot disease represent combining multiple etiologies with differing prognoses. Alternatively, the variation in outcomes may represent variation in clinician training and expertise or differing levels of access to medications and other resources. If this is the case, investments in training and medical resources could improve treatment outcomes. Even where treatment appears to generally be successful, such as for ecto- and endoparasites, improved access to antiparasitic drugs and research demonstrating drug efficacy may improve outcomes.

Causes of morbidity and mortality may adversely affect individual animal health and welfare or have population-level impacts on fecundity and/or mortality rates. Syndromes with mortality rates that exceed morbidity rates may disproportionately affect population dynamics, and thus may warrant greater support for research and interventions. Injuries and infectious disease not due to parasitism may represent a greater impact on elephant mortality than expected (Table 6). However, this study's design precludes definitive conclusions for such comparisons.

## 5. Conclusions

Objective data can assist with prioritizing goals and improving the decision process for maximizing the benefits of health and conservation programs [3, 16]. Results from this questionnaire serve as a starting point for determining how to allocate resources to best benefit elephant health and welfare in Asia, as existing literature is insufficient to support such efforts. While the medical concerns listed by respondents have varying degrees of overlap and should be considered on a coarse scale, trauma was consistently listed as the most common cause of elephant morbidity and mortality. In addition, deficiencies in basic husbandry and diagnostics for elephants were commonly listed. Consequently, captive elephants may benefit most by addressing basic concerns such as nutrition, water availability and quality, and housing. Similarly, minimizing human-elephant conflict for wild populations is a substantial challenge. However, addressing these challenges may yield the greatest health benefits because prevention is often the most cost-effective approach [17].

A focus on one or a few diseases that are not strategically selected risks wasting limited resources by investing in programs that have little or no impact on overall elephant conservation efforts. There is a need to clarify the tradeoffs that exist and establish the outcomes that will likely result from investments in elephant health. Consequently, elephant health programs should be well-supported as a part of overall elephant conservation efforts in Asia.

## Figures and Tables

**Table 1:** Study designs and topic categories that characterize elephant studies conducted in Asia that were identified in a PubMed search.

	Number
Study design	
Ecological or natural history	21
Case report or series	18
Laboratory-based investigation	17
Cross-sectional survey	8
Policy	3
Review	3
Study topic category^*^	
Common disease conditions	31
Basic veterinary concerns	15
Treatment	2
Needs assessment	1

^*^Twenty-one studies could not be categorized by these topics.

**Table 2:** Characteristics of elephant health survey respondents.

Row labels	Number of respondents	Mean number of years of elephant care	Percentage of work with captive elephants (number of respondents)		Percentage of work with wild elephants (number of respondents)	
			0–19%	80–100%	0–19%	80–100%
India	8	9.9	1	3	4	2
Indonesia	21	10.0	1	9	9	1
Laos	2	2.8		1	1	
Malaysia	1	9.0				1
Myanmar	1	10.0				
Nepal	2	13.0		1	1	
Sri Lanka	7	16.4	4	3	2	1
Thailand	3	8.3		2	3	

Grand total (mean ± SD)	45	(10.7 ± 6.8)	6	19	20	5

**Table 3:** Major postmortem pathologic findings reported by survey respondents. Respondents were able to report more than one finding per necropsy.

Reported postmortem pathologic findings	%
Human elephant conflict (electrocution, poisoning, poaching, train collision, gunshot, wells, pit traps, snares, etc.)	10.4
Hemorrhage	10.2
Endoparasites	7.5
Gastrointestinal stasis or torsion	6.7
Lung lesions	6.5
Liver lesions	4.3
Old age	4.1
Undetermined	4.1
Injuries from intraspecific aggression	3.9
Toxin	3.5
Splenic lesions	3.5
Tetanus	3.5
Cardiac lesions	3.3
Renal lesions	3.3
Enteritis	3.1
Traumatic injuries	3.1
Sepsis	2.7
Emaciation	2.4
Skin lesions	2.2
Rabies	1.4
Tuberculosis	1.4
Elephant endotheliotropic herpes virus	1.4
Autolysis	1.2
Cyanosis	1.0
Peritonitis	1.0
Lightning	0.8
Salmonellosis	0.8
Arthritis	0.8
Anemia	0.6
Nasal and oral mucosa ulceration	0.6
Abscess	0.4
Eye conditions	0.4

Grand total	100

**Table 4:** Availability of laboratory facilities reported by survey respondents (*n* = 45).

Diagnostic modality	Always available (number)	Mostly available (number)	Sometimes available (number)	Never available (number)	No response (number)
Hematology	7	17	17	3	1
Organ indices	8	15	16	6	
Blood mineral	4	6	13	22	
Blood nutrition	2		19	24	
Hair nutrition		1	6	38	
Toxin detection		6	17	22	
Hormone analysis	3	1	17	22	2
Fecal parasite microscopy	22	13	9	1	
Blood parasite microscopy	12	8	21	4	
Microbiology culture and antibiotic sensitivity	4	8	26	7	
Methods for identifying viral pathogens	1		14	30	
Histopathology	4	6	26	8	1
Ultrasound	2	4	11	28	
Radiology	1	1	11	32	

**Table 5:** Survey respondents' (*n* = 45) perceptions of needed improvements in the diagnosis, treatment, and preventive medicine for elephants in Asian range countries.

Category of concerns	Specific concerns for each category	Number of respondents indicating a need (%)
Obstacles that prevent obtaining needed medications	Financial constraints	15 (33.3)
	Limited drug availability	29 (64.4)
	Import restrictions for drugs	18 (40)

Obstacles that prevent conducting needed treatments	Limited access to elephants (roads, etc.)	11 (24.4)
	Treatment not permitted by owner	4 (8.89)
	Absence of mahout cooperation	2 (4.44)
	Untrained/uncooperative elephants	16 (35.6)
	Limited diagnostics that could serve to direct appropriate treatments	30 (66.7)

Obstacles to performing diagnostic procedures	Financial constraints	15 (33.3)
	Limited diagnostic test availability	29 (64.4)
	Import restrictions for diagnostic tests	11 (24.4)
	Broken equipment	6 (13.3)
	Lack of personnel training	19 (42.2)
	Shortage of trained personnel	23 (51.1)

Preventive medicine needs: food	Vitamin supplements	33 (73.3)
	Mineral supplements	34 (75.6)
	High nutrient supplements	31 (68.9)

Preventive medicine needs: shelter	Clinic for conducting treatments	25 (55.6)
	Nursery and/or sick elephant facilities	21 (46.7)
	Isolation facility	18 (40)
	Facility for musth bulls	23 (51.1)

Preventive medicine needs: water	Control of H_2_O quality	13 (28.9)
	Control of H_2_O quantity	13 (28.9)

Preventive medicine needs: mahout training topics	Daily routine health	36 (80)
	Elephant restraint and handling tools	28 (62.2)
	Elephant biology and behavior	29 (64.4)
	Drug use and administration	27 (60)

**Table 6:** Major syndromes reported for morbidity and mortality in Asian elephants in range countries, listed in order of most common to least common. Items in ( ) are subcategories of the immediately preceding topic.

Syndromes: morbidity	Syndromes: mortality
Injury	Injury
(Gunshot wounds)	(Gunshot)
Parasitism	Infectious disease not due to parasitism
Gastrointestinal disease	Gastrointestinal disease
(Diarrhea)	(Diarrhea)
Ocular disease	Poisoning
Foot pathology	Old-age related
Malnutrition	Nonspecific
Abscess	Parasitism
Infectious disease not due to parasitism	Malnutrition
Lameness	Renal disease
Skin disease	Cardiac disease
Stereotypical behavior	Hemorrhagic disease
Overwork	Lack of veterinary care
Poisoning	Lameness
Renal disease	Musth
Cancer	Respiratory disease
Ventral edema	Dehydration
Drug reaction	Prolonged recumbency
Reproductive problem	Seizure
Tusk pathology	Congenital disease
	Neurologic disease
	Reproductive problem
	Chemical immobilization

**Table 7:** Syndromes and treatments that are available or are needed and currently unavailable, as well as outcomes with and without treatment.

	Injury	Gastrointestinal disease	Malnutrition	Lameness	Foot diseases	Infectious disease	Parasitic disease	Ocular disease
Standard treatment	Abscesses and myiasis, general and wounds: flush with saline, H_2_O_2_, iodine, chlorhexidine; surgical drainage; topical and systemic antibiotics 7–15 d (oxytetracycline and penicillin); local betamethasone and unspecified anti-inflammatories; conservative compress; vitamins; analgesics; tetanus toxoid; fly repellants; fluid therapy; rest. Gunshot: anesthesia; wound dressing; broad spectrum antibiotic	General: antibiotics; fluids; oral electrolytes; antibiotics (ampicillin, gentamycin); ranitidine; tolterodine; budesonide; metaclopromide; nonsteroidal anti-inflammatories; antispasmotics; vitamins; minerals Colic: enema; fluids and electrolytes; exercise; nonsteroidal agents; bath in pool; antibiotics; anthelmintics; liver extract Constipation/impaction: fluids; rectal palpation; supportive therapy; enemas; purgatives; rest; parasympathomimetics; spasmolytics Bloat: supportive therapy; per rectum neurostimulation; antibloat agents; rectal enemas; exercise; flunixin meglumine. Diarrhea: antibiotics; fluids; diet correction; astringents; antiparasitics; fecal culture. Malabsorption: vitamin and mineral levels in blood; nutritional supplements	Improved food quality; vitamins, minerals, and other food supplements; antiparasitic agents; regular monitoring of serum levels	Nonsteroidal anti-inflammatories; steroids; herbal drugs; hot massage; cool compresses; antibiotics; supportive treatment; rest Fractures: bandage; rest/restriction of movement; plaster cast	Routine foot care; dry location tethering; isolation. Cleaning or bath with H_2_O_2_, iodine or formalin. Local, topical, or systemic antibiotics (tetracycline, broad-spectrum, or based on culture and sensitivity.) Local steroids; supportive therapy	General: antibiotics Tuberculosis: antibiotics (rifampin, ethambutol, or isoniazide) or no treatment EEHV: famcyclovir, acyclovir Tetanus: antispasmodic drugs; antibiotics; hydration; tetanus toxoid Elephant pox: antibiotics; fluids; vitamins; local therapy	General: topical and systemic antiparasitic agents (fenbendazol, albendazol, mebendazol, ivermectin, oxyclozanide); supportive therapy; routine fecal examination Balantidia: no response Trypanosomiasis: Berenil *Cobboldia* spp. and filarial: ivermectin	Ophthalmic topicals: gentamycin, chloramphenicol, nonsteroidal anti-inflammatories, doxycycline, and tetracycline Systemic drugs: nonsteroidal anti-inflammatories Not specified: antibiotics, herbal medications, antifungals, analgesics, glaucoma drugs Surgery

Percentage resolved without treatment (range/number of respondents)^†^	General: 0–80% Gunshot: 50%	General: 1–60% Colic: 0–10% Constipation/impaction: 0–30% Bloat: 0–100% Diarrhea: 0–30% Malabsorption: 0%	0–20%	General: 0–20% (70% for arthritis) Fractures: 0–20%	0–70%	Tuberculosis: 0–30% EEHV: 0–20% Tetanus: no responses Elephant pox: 1%	General: 0–30% Balantidia: 0% Trypanosomiasis: 20% *Cobboldia* spp. and filarial: 10–20%	Conjunctivitis/keratitis: 0–50% Nonspecific ocular disease: 1–40%

Percentage that die without treatment^†^	General: 0–70% Gunshot: 50%	General: 0–70% Colic: 0–80% Constipation/impaction: 0–50% Bloat: 1–1005 Diarrhea: 0-100% Malabsorption: 100%	0–20%	General: 0–5% (40% degenerative joint disease) Fractures: 20–100%	0–50%	Tuberculosis: 0–60% EEHV: 0–80% Tetanus: 100% Elephant pox: 90%	General: 0–50% Balantidia: 0% Trypanosomiasis: 80% *Cobboldia* spp. and filarial: 50–60%	Conjunctivitis/keratitis: Conjunctivitis/keratitis: 0–10% Nonspecific ocular disease: 0–5%

Percentage cured with standard treatment^†^	General: 25–100% Gunshot: 90%	General: 90–100% Colic: 70–100% Constipation/impaction: 60–100% Bloat: 80–100% Diarrhea: 60–90% Malabsorption: no response	100%	General: 70–80% (40% degenerative joint disease) Fractures: 15–60%	30–90%	Tuberculosis: 20–80% EEHV: 25–40% Tetanus: 30% Elephant pox: 100%	General: 80–100% Balantidia: 1000% Trypanosomiasis: 50% *Cobboldia* spp. and filarial: 90–100%	Conjunctivitis/keratitis: 50–100% Nonspecific ocular disease: 40–100%

Percentage mortality with standard treatment^†^	General: 0–10% Gunshot: 10%	General: 0–5% Colic 0–20%: Constipation/impaction: 0–20% Bloat: 0–1% Diarrhea: 0–40% Malabsorption: no response	0%	General: 0% (50% degenerative joint disease) Fractures: 10–85%	0–40%	Tuberculosis: 0–20% EEHV: 25–60% Tetanus: 70% Elephant pox: 10%	General: 0–10% Balantidia: 0% Trypanosomiasis: 50% *Cobboldia* spp. and filarial: 5–10%	Conjunctivitis/keratitis: 0–10% Nonspecific ocular disease: 0%

Treatments that are wished for	General: Suture; minor surgical instruments; antibiotics; drugs that enhance granulation or destroy pyogenic membranes; hoisting facility; topical cream; radiology access	General: routine anthelmintics; probiotics Colic: improved diagnostics, drug efficacy, husbandry records, trochars, diet, and routine anthelmintics; Constipation/impaction: endoscopic surgery Bloat: no responses Diarrhea: improved diagnostics; uncertain Malabsorption: no response	Improved food and supplement resources; reduced corruption of government funds; standardized nutritional guidelines	Improved drugs; improved diagnostic tools; acupuncture; hoisting facility	Elephant training for routine, hygienic foot care and foot care tools; improved staff training; increased enclosure size; cryosurgery; radiology capacity; shoes to prevent wound contamination	General: emphasis on prevention; medications specific for organism Tuberculosis: cheaper drugs with less toxicity and shorter treatment protocols; testing options; EEHV: early diagnostic tools; famcyclovir Tetanus: uncertain Elephant pox: clinical laboratory facility	General: preventive therapy; improved diagnostics and drug efficacy; therapeutic baths Balantidia: no response Trypanosomiasis: less toxic drug *Cobboldia* spp. and filarial: improved laboratory diagnostics and improved mobility to improve response time	Increased variety of drugs available (concerns for antibiotic resistance and efficacy); improved housing (preventive); clinic for morbid animals; ophthalmoscopes and improved diagnostics; improved drug application methods; improved surgical options

Miscellaneous conditions included urinary tract infections, heat stroke, snake bite, photosensitivity, poisoning, ventral edema, septicemia, and pneumonia.

^†^Inconsistencies in numbers (percentages that exceed or are less than 100%) are due to subjectivity and recall bias or language barriers.

**Table 8:** 

Date: —————————————					
(1) Which Asian range country do you work in? ————————————————————					
(a) What region within that country? ———————————————————————————					
(2a) Do you work for a government agency?					
□ No □ Yes If yes, what type?					
□ Forestry					
□ Wildlife					
□ Livestock					
□ Other ——————					
(2b) Do you work for a nongovernment agency (NGO)?					
□ No □ Yes If yes, what type? —————————————————————————					
(2c) If you responded “no” to both (2a) and (2b) please indicate your employment type —————————					
(3) How often do you work with elephant health care?	□ Full time		□ Part time	□ Occasionally	
(4) Approximately how many years have you worked with elephant health care? ———————years					
(5) How many elephants do you check and/or treat on average **each month**? ———————Elephants					
(6) In the area where you are working, how many individual elephants do you see for treatment and/or health check on **a regular basis (minimum 2 times per year)**?	————————————————Elephants				
(7) In the area where you are working, how many of the captive elephants (males and females) have successfully reproduced?	□ All		□ Some		□ None
**What is the percentage of your work with captive or wild elephants**:	0–19%	20–39%	40–59%	60–79%	80–100%
(8a) With captive elephants?	□	□	□	□	□
(8b) With wild elephants?	□	□	□	□	□
**In the area where you are working, what is the percentage of the captive elephants owned**:	0–19%	20–39%	40–59%	60–79%	80–100%
(9a) By the government?	□	□	□	□	□
(9b) By private owners/institutions?	□	□	□	□	□

(10) **For which type of activities are the elephants utilized?**	All of them	Most of them	Some of them	A few of them	None of them

Zoo exhibition	□	□	□	□	□
Tourism	□	□	□	□	□
Logging	□	□	□	□	□
Forest and habitat patrols	□	□	□	□	□
Human elephant conflict management	□	□	□	□	□
Education awareness programs	□	□	□	□	□
Not utilized	□	□	□	□	□

**Table 9:** 

Date: —————————————————					
(11) **On average, how many elephants that you checked regularly (at least 2 times a year) had evidence of the following**:	0–19%	20–39%	40–59%	60–79%	80–100%

No visible signs of diseases or disorders	□	□	□	□	□
Systemic illness	□	□	□	□	□
Upper respiratory disease	□	□	□	□	□
Lower respiratory disease	□	□	□	□	□
Weight loss	□	□	□	□	□
Diarrhea	□	□	□	□	□
Other intestinal problems such as colic or constipation	□	□	□	□	□
Ectoparasites, such as lice, mites, and ticks	□	□	□	□	□
Skin disease caused by reasons other than ectoparasites	□	□	□	□	□
Microscopic detection of infestation with endoparasites	□	□	□	□	□
Shedding endoparasites with feces	□	□	□	□	□
Eye disease	□	□	□	□	□
Oral disease	□	□	□	□	□
Tusk or molar problems	□	□	□	□	□
Trunk disease	□	□	□	□	□
Simple foot problems (overgrown toenails or foot pads, simple cracks or splits in nails and pad without infection and lameness)	□	□	□	□	□
Complicated foot problems (pad and nail infections, serious injuries, pain, swelling, and lameness)	□	□	□	□	□
Non-foot lameness	□	□	□	□	□
Wounds other than skin lesions	□	□	□	□	□
Infant mortality	□	□	□	□	□
Reproductive problems	□	□	□	□	□
Reduced endurance for work	□	□	□	□	□
Reduced appetite	□	□	□	□	□
Stereotypical behavior	□	□	□	□	□
(11b) **Have you conducted any postmortem examinations (necropsies) or witnessed the conduction of these examinations in elephants?**					
□ No □ Yes					
(11c) **If yes, how many cases per year?** ————————————————					
**What were the major pathological findings in these examinations?**					
(“undetermined” can be listed as a finding)					
(1) ——————————————					
(2) ——————————————					
(3) ——————————————					
(4) ——————————————					
(5) ——————————————					
(6) ——————————————					

**Table 10:** 

Date: —————————————————				
(12a) **How often are the following labs and diagnostic facilities available?**	Never	Sometimes	Mostly	Always

Laboratory for conducting **routine hematology**	□	□	□	□
Laboratory for **analyzing blood for routine organ function indices** (e.g., AST, Alk Phos, BUN, Creat, protein, glucose, etc.)	□	□	□	□
Laboratory **for conducting routine blood mineral analyses** (e.g., Na, CL, Ca, P, etc.)	□	□	□	□
Laboratory for **analyzing blood for nutritional indices** (e.g., vitamins, fatty acids, micronutrients, etc.)	□	□	□	□
Laboratory for **analyzing hair for nutritional indices** (e.g., vitamins, micronutrients, etc.)	□	□	□	□
Laboratory for **detection of toxins**	□	□	□	□
Laboratory for conducting **hormone analysis**	□	□	□	□

(12b) **How often are the following labs and diagnostic facilities available?**	Never	Sometimes	Mostly	Always

Light microscopy for **fecal parasite testing**	□	□	□	□
Light microscopy for **blood parasite testing**	□	□	□	□
Microbiology lab for **determination of bacterial pathogens and antibiotic sensitivity**	□	□	□	□
Microbiology lab for **determination of viral pathogens**	□	□	□	□
Histopathology	□	□	□	□
Ultrasonography	□	□	□	□
Radiology (or X-rays)	□	□	□	□

**Table 11:** 

Date: —————————————————					
(13) **Are mahouts regularly screened for any diseases?**		□ Yes	□ No	□ Unsure	

(13a) **Approximately what is the percentage of **	0–19%	20–39%	40–59%	60–79%	80–100%
**mahouts you have seen with the following conditions?**					

Frequent sneezing	□	□	□	□	□
Frequent coughing	□	□	□	□	□
Sudden weight loss	□	□	□	□	□
Eye disease	□	□	□	□	□
Abnormal joints or limbs	□	□	□	□	□
Abnormal skin conditions	□	□	□	□	□
Lethargy (loss of energy)	□	□	□	□	□

**Table 12:** 

Date: —————————————————	
(14) What are the obstacles that **prevent you from** obtaining **needed medicines** (check all that apply)?	
□ Costs for medicines/financial limitations of elephant owner	
□ Limited availability of medicines on the local market	
□ Import restriction for medicines not available on the local market	
□ Other If “other”, please specify————————	
(15) What are the obstacles that **prevent you from** conducting **needed treatments** (check all that apply)?	
□ Limited access to the area where the elephant is located (road conditions, no transportation, etc.)	
□ Treatment not permitted by elephant owner	
□ Mahouts that do not cooperate	
□ Elephants that are not trained/do not tolerate needed treatment procedures	
□ Limited diagnostic techniques to sufficiently diagnose diseases and identify ideal treatment schemes	
□ Other If “other”, please specify————————	
(16) What are the obstacles that **prevent you from** performing **needed diagnostic procedures** (check all that apply)?	
□ Costs for tests/financial limitations of elephant owner	
□ Limited availability of diagnostic tests	
□ Import restriction for diagnostic tests or supplies that limit availability	
□ Inability to keep equipment in working order	
□ Lack of training	
□ Insufficient availability of trained personnel to conduct tests	
□ Other If “other”, please specify————————	
(17) What are the **preventive medicine program needs** that exist (check all that apply)?	
**Elephant food:**	
□ Vitamin supplements	
□ Mineral supplements	
□ Specific food supplements with high nutrients	
□ Other; please specify———————	
**Shelter:**	
□ Special location and facilities for the conduction of treatments (a clinic)	
□ Shelter for sick elephants and/or mothers with calf	
□ Isolation facility	
□ Specific facility/restraint for musth bulls	
□ Others; please specify——————	
**Water:**	
□ Control of water quality	
If yes, please describe techniques/measurements used to control water quality	
————————————————	
————————————————	
□ Control of water quantity provided/made available	
**Medications:**	
What kind of preventive medications are administered?	
(Please list as many as you are aware of)	
——————————————————	
——————————————————	
——————————————————	
**Vaccinations:**	
What are the vaccinations administered? (Please list as many as you are aware of)	
——————————————————	
——————————————————	
——————————————————	
**Mahout training:**	
□ Training about daily routine health care procedures	
□ Training about different restraint and handling tools	
□ Training about elephant biology and behavior	
□ Appropriate use of basic drugs and administering basic medications	
□ Other (if “other,” please specify)	
——————————————————	
——————————————————	
——————————————————	
(18) What kind of preventive programs would you suggest as useful to be conducted in your area, and which currently cannot be conducted? (Please provide details where possible)	
(A) **Elephant food**——————————————————————————————————	
(B) **Shelter**—————————————————————————————————————	
(C) **Water**—————————————————————————————————————	
(D) **Medications**———————————————————————————————————	
(E) **Vaccinations**——————————————————————————————————	
(F) **Mahout management training**——————————————————————————	
(G) **Veterinary training**———————————————————————————————	
(H) **Other**—————————————————————————————————————	

**Table 13:** 

Date: —————————————————			
(19) What are the six main medical issues you find with elephants in your care (i.e., injuries, disease, parasitism, malnutrition, etc.) ranked in order, **from most common to least common?**			
(1) Most common——————————————			
(2) ————————————————————			
(3) ————————————————————			
(4) ————————————————————			
(5) ————————————————————			
(6) Least common——————————————			
(20) What are the six main causes of elephant mortality that you see ranked in order, **from most common to least common?** (“undetermined” can be listed as a cause)			
(1) Most common cause ——————————			
(2) ———————————————————			
(3) ———————————————————			
(4) ———————————————————			
(5) ———————————————————			
(6) Least common cause ——————————			

The following question requests information on the most common disease conditions that you encounter, treatment option used, the treatment results, and ideal future treatment options. Please list the diseases in order from the most common to the least common.			

**Disease:**	(Most Common Disease)		
	(1)————	(2)————	(3)————

**Standard treatment:**			
**Percentage that resolved without treatment:**	————%	————%	————%
**Percentage that died without treatment:**	————%	————%	————%
**Percentage cured with standard treatment:**	————%	————%	————%
**Percentage of mortality with standard treatment:**	————%	————%	————%
**Treatment option(s) that you would like to have available for the future:**			

**Disease:**			(Least Common Disease)
	(4)————	(5)————	(6)————

**Standard treatment:**			
**Percentage that resolved without treatment:**	————%	————%	————%
**Percentage that died without treatment:**	————%	————%	————%
**Percentage cured with standard treatment:**	————%	————%	————%
**Percentage of mortality with standard treatment:**	————%	————%	————%
**Treatment option(s) that you would like to have available for the future:**			

## Appendix

### A. Basic Information

*Survey of Elephant Health and Management in Asia*. This is the first form for the respondents to fill out. The purpose of this form is to obtain basic information about the extent to which the respondent works with elephants. See Table 8.

### B. Basic Veterinary Concerns

*Survey of Elephant Health and Management in Asia*. This questionnaire is a self-report form. The purpose of this form is to obtain percentages of elephants seen by the respondent with select health conditions. See Table 9.

### C. Laboratory Facilities Questionnaire

*Survey of Elephant Health and Management in Asia*. The purpose of this form is to determine the capability of laboratory facilities available for diagnostics. See Table 10.

### D. Mahout Health Questionnaire

*Survey of Elephant Health and Management in Asia*. The purpose of this form is to determine whether the mahouts have any observable health conditions. See Table 11.

### E. Needs Assessment

*Survey of Elephant Health and Management in Asia*. The purpose of this form is to identify common needs and/or barriers to optimal elephant health. See Table 12.

### F. Common Disease Conditions

*Survey of Elephant Health and Management in Asia*. The purpose of this form is to obtain information on common disease conditions encountered and how these diseases are handled by the respondent. See Table 13.
